# Aptamer Cell-Based Selection: Overview and Advances

**DOI:** 10.3390/biomedicines5030049

**Published:** 2017-08-14

**Authors:** Silvia Catuogno, Carla Lucia Esposito

**Affiliations:** Istituto di Endocrinologia ed Oncologia Sperimentale “G. Salvatore”, CNR, Naples 80100, Italy

**Keywords:** aptamer, cell- Systematic Evolution of Ligands by Exponential enrichment, cell targeting

## Abstract

Aptamers are high affinity single-stranded DNA/RNA molecules, produced by a combinatorial procedure named SELEX (Systematic Evolution of Ligands by Exponential enrichment), that are emerging as promising diagnostic and therapeutic tools. Among selection strategies, procedures using living cells as complex targets (referred as “cell-SELEX”) have been developed as an effective mean to generate aptamers for heavily modified cell surface proteins, assuring the binding of the target in its native conformation. Here we give an up-to-date overview on cell-SELEX technology, discussing the most recent advances with a particular focus on cancer cell targeting. Examples of the different protocol applications and post-SELEX strategies will be briefly outlined.

## 1. Introduction

**Figure 1:**
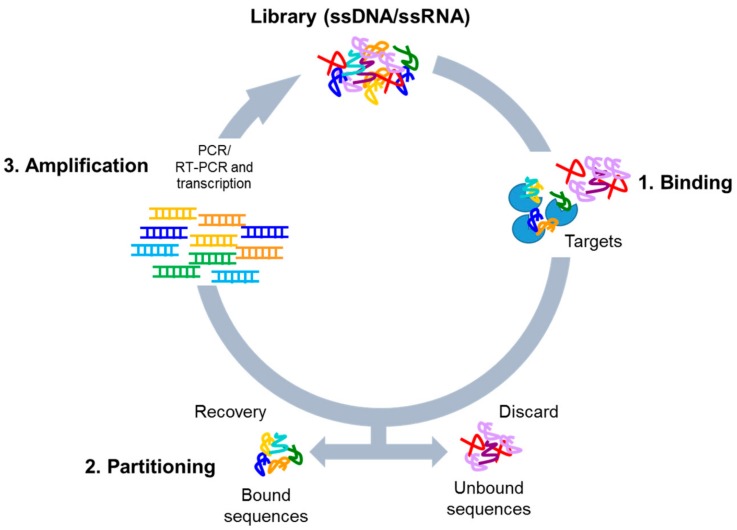
Scheme of the SELEX process. The procedure involves repeated cycles of: **1**. Incubation of the high complexity library with the targets (binding); **2**. Removal of unbound sequences and recovery of the bound oligonucleotides (partitioning); **3**. Amplification of the bound sequences by PCR (for DNA library) or RT-PCR and transcription (for RNA library).

Nucleic acid-based aptamers are single-stranded DNAs or RNAs able to bind with high affinity (dissociation constant in nanomolar/picomolar range) and specificity to a given target by folding in tridimensional structures. They are also called “chemical antibodies” and possess many advantages over antibodies as tools in research, diagnosis, and therapy. Indeed, aptamers are not immunogenic, have small size, a superior chemical stability and versatility, as well as an easy production. Aptamers are selected by Systematic Evolution of Ligands by EXponential enrichment (SELEX) technology, that was introduced for the first time by Tuerk and Ellington about thirty years ago [1,2]. The procedure is based on the isolation of high affinity ligands from a combinatorial single-stranded nucleic acid library through repeated cycles of binding, partitioning, and amplification (Figure 1). Following SELEX cycles, the final aptamer pool is subjected to sequencing for the identification of the best binding sequences.

So far, the SELEX strategy has been applied to a wide range of targets, including purified proteins, small molecules, live cells, tissues, and microorganisms. Traditionally, soluble purified proteins are the most common targets used in SELEX. This strategy continues to be largely applied since it is very simple and can be conducted under well-controlled conditions. However, the selection is performed in a non-physiological context and recombinant proteins often cannot fold into the correct structure. Consequently, it might be possible that selected aptamers failed to recognize their targets in the native conformation. The use of whole living cells (cell-SELEX) as a complex target has the advantage to overcome this limitation, permitting the selection of aptamers in a physiological context. Therefore, differently from protein-based SELEX, during the cell-SELEX procedure all molecules are in their natural folding structure and distribution enhancing the possibility of success in selecting aptamers for in vivo use. For this reason, cell-SELEX has been the first choice to select aptamers against cell surface proteins for both diagnostic and therapeutic application. Another advantage of the cell-based procedures is that they may be employed for the targeting of a specific cell type, without any prior knowledge of the specific target, leading to identify multiple aptamers able to recognize specific cell phenotypes and discover new cell biomarkers. To date, aptamers have been developed for different cell types and other complex systems, especially for live cancer cells, by applying several selection protocols that have undergone progressive changes and improvements.

In this review, we will give an overview of the most important developments of the cell-SELEX methodology, discussing the recent progress with a particular focus on cancer-related aptamers. We will provide examples of aptamer applicability and of post-SELEX modifications that may be introduced into aptamer sequences to improve their in vivo use. Finally, strategies aimed to make the selection process much more rapid and cost-effective will be also discussed.

## 2. Aptamers versus Antibodies

Nucleic acid aptamers act like antibodies and can rival them as cell specific targeting molecules. Indeed, antibodies and aptamers have similar recognition properties with high affinities (in the low nanomolar to picomolar range), but aptamers show a number of important advantages [3]. Indeed, they have superior specificity, simple and cost-effective production, and are resistant to harsh environments (pH and temperatures). Additional advantages of aptamers over antibodies include the possibility to be chemically synthesized once selected, with high purity and very low inter-batch variability, and the easy way by which they may be chemically modified. Modifications (discussed in Section 6) can be done to further improve their stability, bioavailability, and pharmacokinetics, or to allow their simple immobilization or labeling for their use in diagnostic assays. Further, as compared to antibodies, aptamers have smaller sizes (20- to 25-fold as compared with full-sized monoclonal antibodies), allowing improved tissue penetration. Most importantly, they are not immunogenic, thus permitting the safe administration of repeated doses that are precluded for antibodies, unless they are humanized or fully human produced.

## 3. Systematic Evolution of Ligands by EXponential Enrichment (SELEX) Library Design

The starting point of the SELEX procedure is the generation of a single-stranded nucleic acid (RNA or DNA) library with high sequence complexity. Typically, the library contains a random central region of 20–50 bases, whose randomization generates a complex pool of different sequences with different conformations and binding abilities. The dimension of this random region is an important aspect. Indeed, the use of short SELEX libraries (<50 nucleotides) has the advantage of giving shorter aptamers, enabling an efficient and cost-effective chemical synthesis, but at the same time it reduces the library complexity and might limit the effectiveness of the selection. The central part is commonly flanked by two constant regions that are required for PCR amplification and transcription (in the case of RNA library). The length of these primer sequences is an important point in the design of the library, as well. Given the desire to develop short aptamer molecules, there is a growing interest to use libraries with less or no primer sequences. In addition, since the primers may cover more than half of the total aptamer sequence, they may largely influence the library complexity and aptamer-target interaction. Protocols employing shorter conserved sequences (7–10 nucleotides) have been developed [4,5]. Such strategies include the use of self-complementary sequences to sequester the constant regions, thus reducing their influence on aptamer-target interaction. No-primer library selections employing enzymatic reactions have been also proposed [6,7]. For example, Pan et al. used endonuclease cleaving of the double stranded DNA to remove primers before each SELEX cycle and a ligation with primer sequences to reconstitute the library after the selection [6]. Even if these procedures are more laborious and not yet applied to cell-SELEX, they may provide interesting alternatives to the standard protocol.

Another factor to consider in the library design is the choice between RNA or single-stranded DNA aptamers. In general, the amplification protocol for a DNA library is simpler than for a RNA-based pool, and DNA oligonucleotides are more stable and cost-effective. On the contrary, RNAs have several advantages as compared to DNAs. First, RNA molecules are more flexible allowing a higher complexity of folding. In addition, RNAs may be easily modified to reach a stability comparable or even better than ssDNAs. So far, both RNA and DNA libraries continue to be used and no significant differences between DNA or RNA aptamer specificity and affinity have been reported [8].

## 4. Cell-Based SELEX Methods

### 4.1. Whole-Cell SELEX Strategy

**Figure 2:**
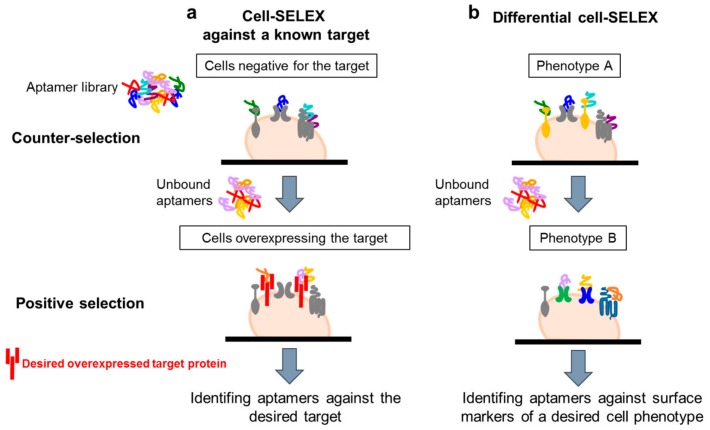
Cell-SELEX variants. Scheme of the two main variants of cell-SELEX: (**a**) protocol to select aptamers against a known target by using in the selection step a cell line over-expressing the target; (**b**) protocol to identify multiple ligands specifically recognizing a cell phenotype, without prior knowledge of the target protein.

The use of complex target for aptamer selection was introduced for the first time by Morris et al. [9] in 1998. They used human red blood cell membrane preparations (RBC ghosts) instead of purified membrane proteins to isolate multiple ssDNA molecules with high affinity for different cell membrane targets. This work opened the path to the development of whole, living cell SELEX protocols. Generally, these protocols have been adopted to isolate aptamers able to recognize a known target of interest or a specific cell phenotype (Figure 2a,b). In both cases, a fundamental aspect is the inclusion of a counter-selection step to avoid the parallel enrichment of aptamers for unwanted targets. The negative selection step is introduced before the positive selection at each round, allowing filtering out sequences against those molecules commonly expressed on both the target and control cell lines.

Many cell-SELEX strategies have been used to isolate aptamers against a known protein of interest by using in the positive selection step a cell line over-expressing the target (Figure 2a). For example, aptamers that specifically recognize the transforming growth factor-β type III receptor (TGFβRIII) have been generated by such an approach [10]. The authors used Chinese hamster ovary (CHO) cells ectopically expressing TGFβRIII as target and isolated a RNA aptamer with a very good dissociation constant (1 nM range), able to efficiently recognize the cell surface receptor. The aptamer is also able to inhibit the interaction between TGFβRII and TGF-β2 ligand in vitro, showing potential applicability as a tool to investigate the receptor function and as therapeutic agent.

This strategy employs mock cells for counter-selection. A novel selection protocol, referred as “Isogenic cell-SELEX” (Icell SELEX), has been recently proposed for the isolation of aptamers against a known target [11]. The authors used HEK293 cells in which the integrin alpha V (ITGAV), a transmembrane receptor expressed in almost all cell lines, is depleted (in the counter-selection step) or over-expressed (in the positive selection step). By such an approach, they easily isolated several anti-ITGAV aptamers.

A variant of the cell-SELEX approach, called hybrid- or crossover-SELEX, has been also proposed in order to enhance the success of the screening and the aptamer specificity for the target, by switching during the selection from cell-SELEX to purified protein SELEX. Such an approach revealed to be effective in isolating two high-affinity Tenascin-C (TNC) aptamers [12]. The adapted protocol included a first enrichment of the pool on U251 glioblastoma cells over-expressing TNC, followed by a further enrichment against the recombinant purified protein. An inverted two-stage selection scheme, combining a first selection against the recombinant protein and a subsequent enrichment by cell-SELEX, has been as well developed to isolate anti-transferrin receptor (TfR) aptamers [13]. The minimized anti-TfR aptamers were used to functionalize stable nucleic acid lipid particles (SNALPs), containing targeted siRNA achieving enhanced and specific uptake and silencing. A dual SELEX protocol was also applied to isolate DNA aptamers targeting the G-protein-coupled cholecystokinin B receptor (CCKBR) that is constitutively expressed by pancreatic ductal adenocarcinomas (PDACs) [14]. The adapted selection strategy combines cycles against “exposed” CCKBR peptides and CCKBR-expressing PDAC cells. Among the isolated sequences, one aptamer (termed AP1153) shows a Kd of 15 pM and is internalized into target cells in a receptor-mediated manner, efficiently acting as carriers of fluorescent nanoparticles (NPs) to PDAC tumors in vivo. Similarly, Martínez Soldevilla et al. [15] used a hybrid SELEX to isolate aptamers targeting the Multidrug Resistant-associated Protein 1 (MRP1), a 17 trans-membrane protein that has been correlated with resistance to chemotherapy in several cancers. The developed protocol includes 10 rounds of selection against a MRP1-peptide and a last round of cell-based selection using the chemotherapy-resistant H69AR cell line, showing a high MRP1 expression, as target. The selected aptamer was then used to generate a MRP1-CD28 bivalent aptamer to deliver the CD28 costimulatory signal of tumor-infiltrating lymphocytes to MRP1-expressing tumors. The bi-specific aptamer enhances costimulation in chemotherapy-resistant tumors and reduces tumor growth in melanoma-bearing mice upon systemic administration. An interesting combinatory screening in vitro and in vivo has been more recently proposed by Zhu et al. [16] to isolate human epidermal growth factor receptor 2 (HER2)-targeting DNA aptamers for preclinical HER2 imaging in ovarian cancer. The authors first performed an in vitro protein-based SELEX on HER2 purified extracellular domain, and then used the resulting DNA pool for a cell-based selection on HER2- over-expressing SKOV3 cells. The best candidates have been radiolabeled with ^18^F and screened in vivo in mice bearing SKOV3-tumors by PET imaging. This strategy led to isolate two aptamers enabling a rapid visualization of HER2-positive tumors with a good tumor uptake and tumor-to-muscle ratios.

An alternative cell-SELEX strategy (referred as “differential cell- SELEX”) (Figure 2b) has been developed to isolate aptamers able to recognize a specific cell phenotype, rather than a single specific target of interest. This strategy offers the possibility to select multiple ligands discriminating between even closely related cell types, without any prior knowledge of the target. Briefly, the procedure consists of the incubation of the starting library on a non-target cell line (with undesired phenotype, negative selection step) followed by the recovery of unbound oligonucleotides that are, then, incubated on cells with the desired phenotype (positive selection step). By using such an approach, ssDNA aptamers able to specifically distinguish T-cell acute lymphocytic leukemia (ALL) cells from B-cell lymphoma cells [17] or small lung cancer cells versus large cell lung cancer [18] have been selected. A similar strategy has been used to isolate aptamer-based probes selective for colorectal cancer cells [19] or nasopharyngeal carcinoma (NPC) [20]. In the last study, the authors performed a cell-SELEX on NPC 5-8F (target cells) using nonmalignant human nasopharyngeal (NP) epithelial NP69 cells (non-target cells) in the counter-selection step. They isolated four aptamers able to discriminate between NPC and NP cells and then employed an aptamer-based affinity purification combined with mass spectrometry, identifying CD109 as the target of the S3 aptamer.

More recently, Yoon et al. [21] applied a “blind” SELEX protocol to pancreatic cancer using Huh7 (hepatocarcinoma cells) as negative cells and PANC-1 cells as positive selection. By chromatography tandem mass spectrometry, the authors demonstrated that one of the aptamers (P15) binds the intermediate filament vimentin, a biomarker of epithelial–mesenchymal transition, and significantly inhibits cell invasion. In addition, the differential cell-SELEX strategy has been applied to discriminate between cell types with different properties (i.e., tumorigenesis and metastatic potential). In this context, our laboratory reported the isolation of a panel of aptamers able to bind human malignant glioblastoma (GBM) cells, discriminating them from non-tumorigenic GBM cells [22]. More recently, Li et al. proposed metastatic-cell-based SELEX to isolate aptamers specific for metastatic cancer cells. The authors applied this strategy to colon cancer, using as target of the selection SW620 cells, derived from metastatic site lymph node. By such an approach, they identified an aptamer (XL-33-1) with high affinity and selectivity for target cells. [23]. Most importantly, tissue imaging experiments with FAM-labeled XL-33-1 revealed its ability to specifically identify the metastatic tumor or lymph node tissues, thus showing its great potential as a molecular imaging agent for early detection of colon cancer metastasis. Similarly, Yuan et al. [24] used metastatic colorectal carcinoma cell line LoVo as selection target and non-metastatic colorectal carcinoma cell line SW480 for the counter-selection, identifying a new aptamer (J3) able to recognize a metastasis-related membrane protein. This aptamer was labeled with Cy5 and used as effective imaging contrast for colorectal carcinoma metastasis with a detection rate of 73.9%. Aptamers able to discriminate high metastatic versus low/non metastatic cells derived from prostate cancer or hepatocellular carcinoma, have been also recently selected by cell-SELEX [25,26]. Selected aptamers were labeled and successfully used for metastatic tissue imaging. Then, Kim et al. [27] proposed an interesting differential protocol, performing repeated cycles of positive selection on tumor-initiating cells (TICs) and negative selection for non-TICs and human neural progenitor cells. This strategy led to isolation of a pool of sequences able to specifically bind to and internalize into TICs, thus showing a potential applicability for TIC targeting and imaging. Further, a DNA aptamer (MS03) specifically recognizing mammospheres was isolated following 13 rounds of selection on MCF-7-derived mammospheres. In the proposed protocol, normal breast epithelial MCF-10A and Salinomycin-treated MCF-7cells were used in the counter-selection step [28]. The authors demonstrated that MS03 aptamer was effective in isolating breast CSCs by flow cytometry. More recently, a modified cell-SELEX method has been applied also to stem-enriched cancer cells in pancreatic cancer by performing positive selection on spheres derived from pancreatic cancer cell line, HPAC, and negative selection on pancreatic normal cell line, HPDE. The approach led to the isolation of two sequences with high specificity and good affinity for the CSC population [29]. Notably, one of the sequences (labeled with Cy5) allows detection of CSC marker over-expressing circulating tumor cells, isolated from blood samples of metastatic pancreatic cancer patients.

In order to investigate cancer cell drug resistance, Zhang et al. [30] applied a differential SELEX protocol using doxorubicin-resistant MCF-7R breast cancer cell line as target of the selection and its parental cell line (MCF-7) for counter-selection. Interestingly, by such an approach the authors isolated an aptamer (M17A2) able to recognize intercellular connections (tunneling nanotubes), thus representing a novel probe for cell-cell communication studying.

All these differential cell-SELEX approaches have the potential to provide multiple targeting ligands with a great applicability as specific probes and tools for biomarker discovery. Nevertheless, a limit arises from the absence of notion of the target recognized by the selected aptamers. Therefore, subsequent target identification strategies based on proteomics analysis [31,32], which are often very complicated and time-consuming, are required.

### 4.2. Cell-SELEX Variants

Currently, several additional new screening methods have emerged to optimize the selection process, some interesting examples are briefly describe below.

#### 4.2.1. Fluorescence-Activated Cell Sorting (FACS)-SELEX

**Figure 3:**
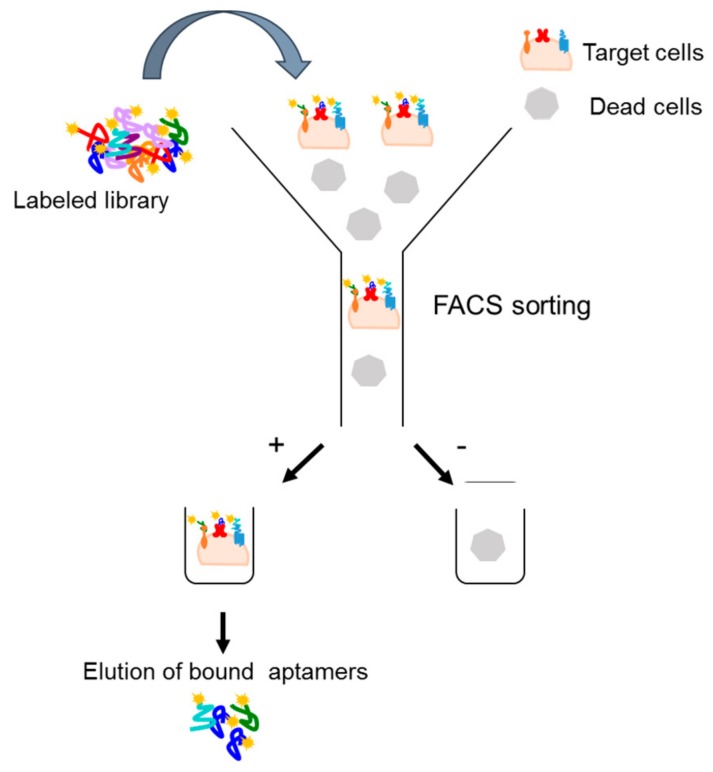
Scheme of FACS-based SELEX. Fluorescently labeled aptamer library is incubated on target cells. A cell-sorting device is used to isolate aptamer-target cells subpopulations and then bound aptamers are eluted for the following cycle.

An extension of the cell-SELEX strategy is a fluorescence-activated cell sorting (FACS)-based protocol that allows to select aptamers targeting a specific subpopulation [33]. In this strategy, once a fluorescently labeled aptamer library is incubated on target cells, a cell-sorting device is used to differentiate and separate the cell subpopulations that are bound or unbound to the aptamers. Bound aptamers are, then, eluted and amplified (Figure 3). The protocol permits to eliminate dead cell population that, absorbing single-stranded nucleic acid molecules, may negatively influence the selection procedure. In addition, such an approach has two additional advantages. First, it allows the reduction of experimental steps, incorporating in one round both positive and negative selection. Second, it allows to simultaneously monitor the selection process during the rounds without the need of additional binding assays. The FACS-SELEX strategy has been, for example, successfully applied by Kim et al. [34] to select an aptamer (EP166) against the transmembrane protein EpCAM that is over-expressed on the surface of most adenocarcinomas and cancer stem cells. The EP166 aptamer can distinguish cells expressing EpCAM from negative control cells and can bind to the mouse embryonic stem cell line J1ES, thus providing a novel stem cell probe.

#### 4.2.2. Cell Internalization SELEX

**Figure 4:**
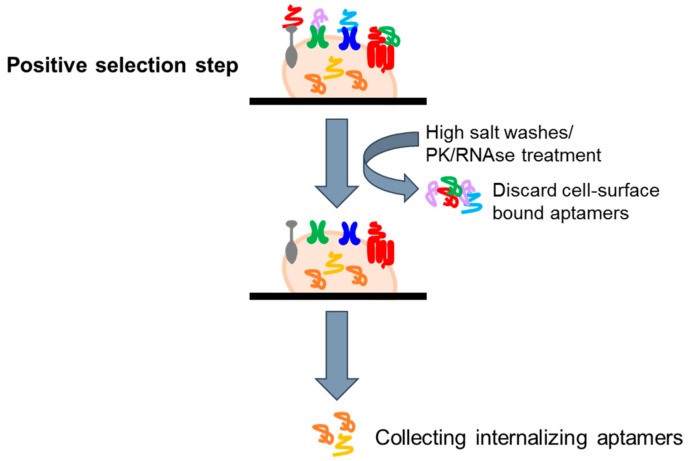
Scheme of protocols to isolate cell internalizing aptamers. Following the selection step, high salt washes or PK treatment are performed to remove cell surface bound aptamers and recover internalized sequences.

In recent years, aptamers are emerging as one of the most promising tools for the specific deliver to diseased cells of secondary reagents. Indeed, it has been shown that upon binding to their targets, aptamers can be rapidly internalized, allowing the tissue specific internalization of active therapeutic substances, including nanoparticles [35,36], anti-cancer therapeutics [37], toxins [38], enzymes [39], radionuclides [40], viruses [41], small interfering RNAs (siRNAs) [42,43,44,45,46,47,48,49,50,51,52,53,54], microRNAs, and anti-microRNAs [55,56,57]. This permits the exposition to secondary reagents only of target cells, increasing the efficacy and reducing the toxicity of the therapy. Based on these considerations, several groups have attempted to develop modified cell-based selection approaches to isolate internalizing aptamers, eliminating those sequences that do not, or very slowly, internalize (Figure 4). In this regard, Thiel et al. [53] proposed a cell-internalizing protocol based on a stringent high salt wash step to remove cell surface bound aptamers and recover only the internalized sequences. The protocol has been applied to a cell line over-expressing HER2, a transmembrane receptor over-expressed in breast cancer and associated with a poor prognosis. The authors successfully isolated RNA aptamers with high specificity for HER2-expressing breast cancer cells and with a good internalization rate. These molecules were efficiently used for the delivery of therapeutic siRNAs to HER2-expressing breast cancer cells. A variant of internalizing SELEX has been then developed in our laboratory [58]. The library coming from 13 rounds of differential cell-SELEX on U87MG cells [16] were subjected to 2 more rounds of a cell-internalization SELEX protocol based on a proteinase K (PK) treatment to remove surface-bound aptamers. The adapted strategy was aimed to preserve the glioma specificity and isolate a pool of sequences with a rapid internalization rate into GBM target cells. By such an approach, we isolated a novel internalizing aptamer (GL53) that was demonstrated to target the insulin receptor.

The use of an RNAse cocktail to remove surface-binding aptamers and recover cell-internalizing aptamers was more recently proposed [59]. Further, an interesting protocol to successfully enrich for aptamers that internalize via the endosomal pathway has been proposed by Ranches et al. [60]. The proposed strategy is based on the isolation of the endosomal fractions by cell homogenization, followed by sucrose gradient centrifugation at each selection cycle.

#### 4.2.3. Ligand-Guided Selection (LIGS)

**Figure 5:**
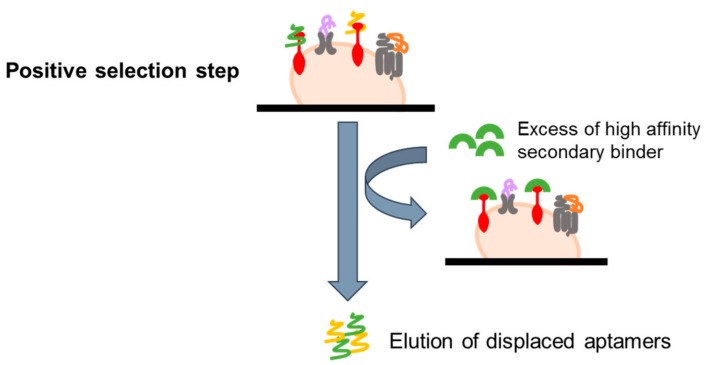
Scheme of Ligand-Guided Selection (LIGS). An excess of high affinity secondary binder (i.e., a monoclonal antibody) for the chosen target is added following the selection step, in order to displace and elute bound aptamers by competing with them.

In order to select aptamers against known epitopes of interest, a variant of the basic live cell-SELEX (referred as ligand-guided selection, LIGS) has been recently developed. The protocol is based on the use of a high affinity secondary binder for the chosen target, such as a monoclonal antibody, that can compete with bound aptamers, displacing them and allowing their elution (Figure 5). In the first report, this approach has been successfully used to select aptamers against two markers on B-cell lymphoma and T cell leukemia, IgM and CD3, respectively [61,62]. The LIGS approach expands the capabilities of cell-SELEX, introducing a very simple method for a selective elution of bound aptamers. Nevertheless, its use is limited to known markers for which high-affinity secondary ligands are available.

#### 4.2.4. Microfluidic-Based System

**Figure 6:**
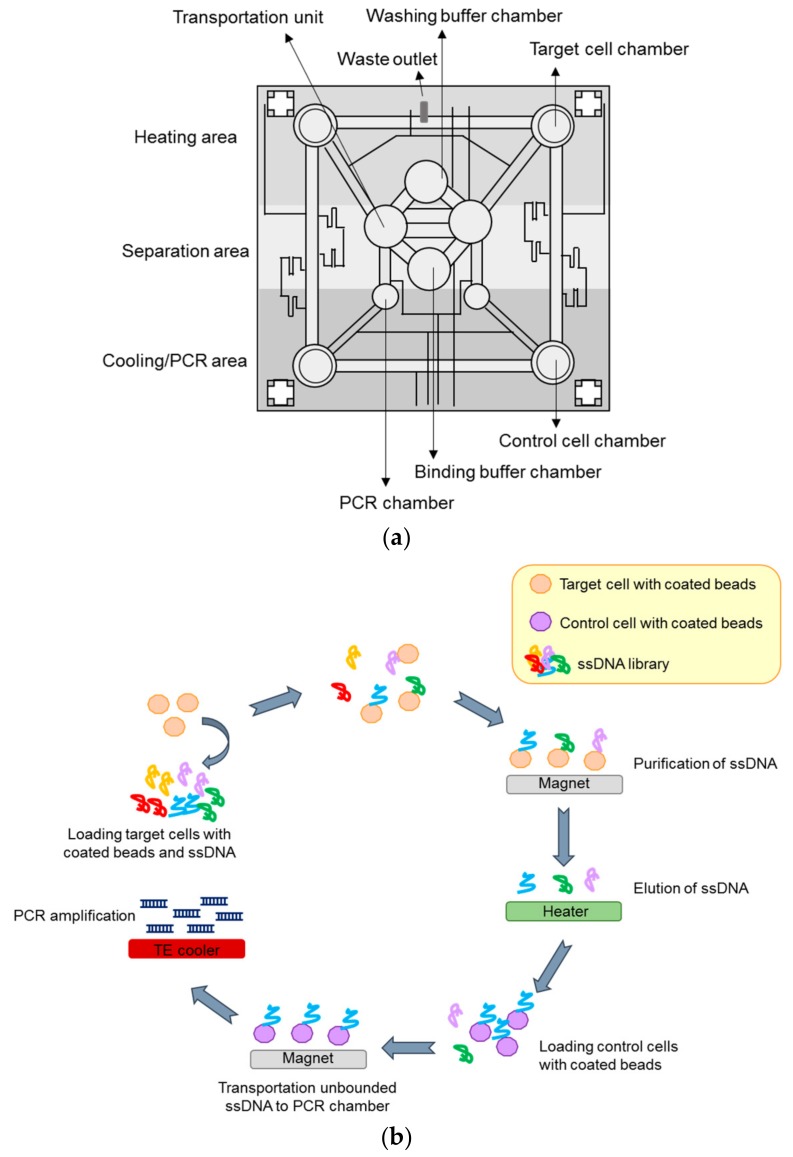
(**a**) Schematic representation of the magnetic-bead-based microfluidic chip used in microfluidic-based cell-SELEX procedure; (**b**) Fundamental steps of the procedure. Aptamers are mixed with magnetic beads-coated target cells (positive selection) and the magnetic-bead complexes are recovered by applying a magnetic field. Bound ssDNAs are then mixed with magnetic beads-coated control cells (negative selection). Control cells–aptamer complexes are collected and the supernatant are transported to the PCR chamber for amplification.

The SELEX-based screening method is generally time-consuming and one challenging aspect is the automation of the method. In this regard, selection processes based on microfluidic chips have been proposed to render the SELEX more rapid and efficient [63,64,65,66]. These technologies have been mostly applied to protein-based selection and, only more recently, the Lee group reported a novel microfluidic system capable of performing automated cell-SELEX [67,68]. The microsystem used comprises three major modules: 1. a microfluidic control module, 2. a magnetic bead-based aptamer extraction module and 3. a temperature control system to regulate the reagent temperature and allow nucleic acid amplification by on-chip PCR. One vacuum pump is employed in the system to control the sample/reagent flux. Briefly, the procedure (Figure 6) starts with the surface coating of target and control cells with the magnetic beads and the loading of all the required reagents (coated cells, library, and buffers) in the storage chambers. During each cycle, the ssDNA library is transported into the incubation chamber and mixed with the magnetic bead-coated target cells (positive selection). The unbound sequences are drawn away and after washings, the magnetic-bead complexes are recovered on the bottom of the incubation chamber by applying a magnetic field. The purified cells are then subjected to thermal lysis and bound ssDNAs are transported to the incubation chamber and mixed with magnetic beads-coated control cells (negative selection). Once magnetic beads control cell-aptamer complexes are collected, the supernatant containing sequences that bind to positive selection but not to the control cells, are transported to the PCR chamber. Here, sequences are amplified for the subsequent round. The proposed approach was successfully used to isolate an aptamer specific for colorectal cancer cells and stem cells [67]. In addition, a similar strategy was applied to cholangiocarcinoma (CCA) cells, leading to isolation of three specific aptamers (one for SNU-478 cells and two for HuCCT-1 cells) [68]. The procedure is completely automated and requires very small volumes and time (only 100 min/round), as compared to standard protocols.

### 4.3. 3D Cell-SELEX and In Vivo SELEX

One of the drawbacks of cell-based selection strategies is that it may happen that in vitro selected aptamers fail to be effective in vivo. Indeed, it is possible that protein target conformation is affected by the target’s environment that can change in vivo. In order to mimic the microenvironment in vitro, recently Souza et al. [69] developed a novel cell-based strategy by using spheroid cells of human prostate cancer in 3D cell culture as target of the selection. The authors performed a first round of negative selection against the non-tumor cell line RWPE-1, followed by eight rounds of selection against PC-3 cell line. By such an approach, they generated an aptamer able to specifically bind prostate tumor cells, with a dissociation constant in the nanomolar scale and potential application in screening assays for prostate cancer.

Most importantly, even very complex targets, including tumors implanted in mice (in vivo-SELEX), have been used to select aptamers. For example, Mi et al. [70] screened a nuclease-resistant RNA library in mice-bearing murine CT26 colon carcinoma liver metastases, identifying an aptamer that binds to p68, an RNA helicase upregulated in colorectal cancer. The same group has also further refined the developed strategy in order to develop a more useful reagent for human patients [71]. As the target they used intrahepatic immunodeficient mice engrafted with two patient-derived cell lines (designated as 119X and 57X) from liver metastases. One of the selected molecules binds DHX9, an RNA helicase upregulated in colorectal cancer. As assessed by fluorescence molecular tomography imaging, this molecule is able to preferentially localize into the nucleus of the cancer cells in vivo following systemic administration, thus showing a potential use for targeted delivery. An additional in vivo selection strategy has been recently proposed to isolate aptamers able to cross the blood-brain barrier (BBB), the brain physiological barrier that protects the brain limiting at same time the therapeutic interventions in neurological disorders [72]. The selection was developed by performing 14 rounds in which a 2 -fluoropyrimidine (2′-F-Py)-modified RNA library was administered to wild-type mice via tail vein injection and brains were harvested for aptamer recovery and amplification. Notably, among the isolated sequences, the authors found some aptamers able to enter brain endothelia and parenchymal cells after peripheral injection.

Despite their sophistication, these in vivo approaches have the great advantage that they can perform the selection in a natural physiological environment. In addition, aptamers are isolated based on their localization, permitting a “built in” negative selection, directly eliminating those sequences that disperse to organs/tissues not of interest.

## 5. Sequencing and Bioinformatic Analysis

At the end of the selection process, a pool of oligonucleotides enriched for aptamers able to recognize the target is obtained and must be further processed to analyze individual candidate sequences. Conventionally, the final library is subjected to PCR-amplification and cloning followed by Sanger sequencing of a small number of positive colonies (50–100) which leads to identifying the most frequent sequences. By using bioinformatics tools, individual aptamers can be: 1. aligned in order to identify identical/similar sequences and group them in clusters [73]; 2. analyzed for their secondary structure to have indications on relevant structures for binding [74,75,76]. The use of high-throughput sequencing (HTS) has opened a new path in the aptamer field, allowing to obtain hundreds of millions of reads from multiple rounds of a selection [77,78,79]. HTS enables a broader and more accurate analysis of the obtained aptamers, giving information on their abundance along with the identification of functional and rare motifs. It also allows study of the evolution of each oligonucleotide population during the selection process. Nevertheless, given the vast amount of data generated, more sophisticated bioinformatic methods are required to identify the best candidates among the hundreds of millions of sequences obtained. Bioinformatic analyses include the processing of HTS data to remove adapter/barcode/constant region sequences, the cutoff and counting of the number of identical reads, and the filtering of the sequence clusters. These processes may be time consuming and almost impractical for many researchers. In this regard, a powerful tool comes from Hoinka et al. [80]. They developed a new open-source platform (referred as AptaGUI) for the dynamic visualization of HT-SELEX data by applying AptaTools package [81]. AptaTools includes algorithms for HTS data preprocessing and allows tracking of the changes of individual aptamers and clusters, to perform structure-based analysis and identify the best candidate sequences. Very recently, Thiel and Giangrande [82] have adapted Galaxy Project tools [83,84], originally developed for HTS genome, exome, and transcriptome dataset analyses, to HTS aptamer data. The authors described simple methods to use tools within Galaxy to pre-process aptamer HTS data, without the need of in-depth computational expertise/knowledge. Since aptamer-target interaction depends on aptamer folding, Pei et al. developed a novel framework to analyze HT-SELEX data that take into account both sequence and structure components allowing identifying structure motifs enriched or depleted during the selection process [85].

## 6. Post-SELEX Modifications Optimization

Once selected, aptamers may be optimized for in vivo applicability in order to: 1. reduce their length; 2. improve their properties in terms of stability and clearance.

A critical aspect for aptamer translation to clinic is the necessity of an efficient and cost-effective chemical synthesis. Long RNA sequences (>60–70 nt) remain difficult to synthesize and have high costs of manufacturing. On the other hand, the use of short SELEX libraries (<50 nucleotides) reduces the library complexity, limiting the effectiveness of the selection [86]. Therefore, longer aptamers are generally selected and then reduced in their length to minimal functional sequences. The most useful approach to achieve this goal is to adopt a “rational truncation” of the aptamer guided by its structural prediction [87]. Briefly, several short sequences are synthesized in order to maintain predicted structural elements and tested for their binding ability. In addition, it has been shown that information derived from the tertiary structure prediction and docking modelling may be useful to reveal key nucleotides for aptamer binding.

In addition to length, aptamer properties may be easily improved. Indeed one main advantage of aptamers is that they can be modified in order to optimize their in vivo stability and persistence in biological fluids. Selections performed with RNA aptamers generally employed libraries containing the substitution at the 2′-ribose of the pyrimidines with 2′-fluoro and 2′-amino groups that greatly enhance nuclease resistance of RNA aptamers [88,89]. A special mutant form of the T7 RNA polymerase (Y639F) is available that allows the introduction of these modifications in the transcription reaction during the selection process [90]. Moreover, other modifications may be introduced in the aptamer sequences by chemical synthesis post-selection. A further increase of nuclease resistance can be achieved by introducing 2′-*O*-Metyl purines [91], phosphorothioates, the capping at the oligonucleotide 3′-terminus [92], or locked nucleic acids (LNAs). LNAs contain a methylene bridge to connect the 2′-*O* to the 4′-C that increases the thermo-stability of oligonucleotides [93], enhancing the resistance to nucleases. An alternative is represented by next generation aptamers, called spiegelmers, developed by Noxxon Pharma. They are artificial oligonucleotides containing L-ribose instead of its natural counterpart, d-ribose [94], thus showing high physico-chemical stability and resistance to all types of nucleases.

Other modifications have been developed to increase aptamer clearance. Indeed, an aptamer’s low molecular weight allows cost-effective chemical synthesis and good target accessibility, but facilitates rapid renal filtration. The most common used modification to reduce this effect is the conjugation with polyethylene glycol (PEG) [95] to increase the aptamer size.

All the described modifications greatly improve aptamer applicability. One concern that needs to be underlined is that the employment of post-SELEX modifications can affect an aptamer’s affinity to its target. Therefore, aptamer binding ability need to be monitored following the introduction of each modification.

## 7. Clinical Applications of Aptamers

Aptamers selected by cell-SELEX show high affinity and specificity for their targets and excellent features for their development as diagnostic tools. Indeed, aptamers can efficiently distinguish between normal and tumor tissues, as well as between different tumor types. In addition, as discussed, nucleic acid aptamers show the advantage of predictable secondary structure and easy chemical modification. Therefore, they can be functionalized with fluorescent probes or nanoparticles for in vivo imaging. Differently labeled aptamers have been developed as innovative tools for magnetic resonance, computed tomography, positron emission tomography, and optical imaging, revealing excellent diagnostic sensitivity for accurate and early diagnosis.

Of note, a vascular endothelial growth factor (VEGF) receptor 2-specific aptamer was conjugated with magnetic nanocrystals for glioblastoma diagnosis through magnetic resonance imaging (MRI) and tested both in vitro and in vivo in glioblastoma-bearing mice [96]. Moreover, an aptamer targeting tenascin-C protein, a biomarker over-expressed on different tumor types, was conjugated with carbon nanodots for optical imaging of cervical cancer [97]. In another study, an anti-mucin (MUC) 1 DNA aptamer, conjugated through phosphorothioate linkers with quantum dots (QDs) and optimized for in vitro and in vivo imaging, was described. The generated molecule exhibited increased photo-stability and reduced toxicity, compared to QDs alone, resulting in strong fluorescence capability in xenograft mouse models [98].

Aptamer-conjugated gold nanoparticles (Apt-AuNPs) have been also generated and used for a colorimetric assay for rapid, simple, direct, and sensitive detection of cancer cells [99]. In addition, using the two-photon scattering (TPS) technique, Lu et al. [100] developed multifunctional (monoclonal anti HER2/c erb 2 antibody and S6 RNA aptamer) AuNP conjugates in which oval-shaped instead of spherical AuNPs were used. This approach proved improved sensitivity for detection of the SK BR 3 breast cancer cells.

Conjugation of aptamers with radioisotopes to develop computed tomography imaging probes has been also achieved. Anti-nucleolin aptamer-functionalized, ultra-small, monodisperse silica nanoconjugates labeled with ^64^Cu radioisotope have been generated and tested in vivo for the identification of lymph nodes in metastatic tumors [101]. In a more recent report, aptamers targeting the human epidermal growth factor receptor were tethered with hollow gold nanospheres (HAuNS) through complementary DNA linkers and labeled with ^111^In. In in vivo mouse models, this molecule demonstrated a greater uptake than ^111^In-labeled antibodies conjugated to HAuNS [102].

From a therapeutic point of view, aptamers with inhibitory capacity on their target can be used to modulate cellular processes associated with human diseases. In addition to the high affinity and specificity, several interesting properties (i.e., low immunogenicity, low toxicity, high batch fidelity and good serum stability) make aptamers ideal therapeutic molecules.

The most successful example of therapeutic aptamer is pegaptanib (Macugen^®^), an anti-VEGF aptamer approved by the U.S. Food and Drug Administration for the treatment of wet age-related macular degeneration. Other aptamers have recently entered clinical trials for the treatment of different human pathologies (Table 1).

Very interestingly, aptamers directed against cell surface epitopes show the ability to internalize in a target-mediated manner and may be used to drive a secondary reagent exclusively to the cell population over-expressing the aptamer target. This is a very attractive strategy in the medical research field, in order to reduce the overall toxicity of treatments.

A growing number of selective delivery strategies by using cell-specific aptamers as carrier molecules have been proposed [112], revealing this approach as a powerful tool for the safe and effective management of different human pathologies.

## 8. Conclusions

From its first application, the cell-based SELEX strategy has been successfully applied to generate aptamers against several cell surface targets. Considering aptamer versatility and features (cost-effective chemical synthesis, easy and controllable modification, nontoxicity), many of these oligonucleotides have great potential as prognostic, diagnostic, and therapeutic tools. In addition, the recent advances in the selection technology have opened the possibility for isolating aptamers for a specific cell phenotype, a particular subpopulation, or even in vivo localization. These molecules also represent promising tools for novel biomarker discovery. However, the clinical translation of cell-based selected aptamers is still in the early stages. Indeed, despite their great potential, several challenges still need to be addressed to realize their use. In recent years much effort has been devoted to accelerating the SELEX process, developing novel, automated, or high-throughput systems, but these strategies have difficult applicability to cell-based selections. In addition, given the complexity of the target, the development of aptamers to be used as a molecular fingerprint of different stages/subtypes of disease still need wider screening methods as well as labeling techniques and tracing studies.

## Figures and Tables

**Table 1:** Aptamers in clinical trials.

Aptamer Name	Composition	Medical Condition	Current Phase	Sponsor	Reference
Pegnivacogen	RNA with 5′-PEG and 3′ inverted dT	Coronary artery disease	Phase III completed	Regado Biosciences	[103]
E10030	DNA with 2′-*O*-methyl, 5′-PEG, 3′ inverted dT	Wet age-related macular degeneration	Phase III	Ophthotech Corporation	[104]
Zimura	RNA with 5′-PEG, 3′ inverted dT	Dry age-related macular degeneration	Phase II/III	Ophthotech Corporation	[105]
AS1411	DNA	Renal cell carcinoma	Phase II	Antisoma Research	[106]
NOX-E36	L-RNA with 3′-PEG	Type 2 diabetes mellitus and albumenuria	Phase II completed	Noxxon Pharma AG	[107]
NOX-A12	L-RNA with 3′-PEG	Chronic lymphocytic leukemia	Phase II	Noxxon Pharma AG	[108]
EYE001	RNA with 5′-PEG	Wet age-related macular degeneration	Phase I completed	National Eye Institute	[109]
ARC19499	DNA with 5′-PEG and 3′ inverted dT	Hemophilia	Phase I completed	Baxalta US Inc.	[110]
NOX-H94	L-RNA with 5′-PEG	Anemia of chronic inflammation	Phase I completed	Noxxon Pharma AG	[111]

PEG, Polyethylene glycol.
